# 761 Albumin to Alkaline Phosphatase Ratio in Burn-Induced Liver Damage

**DOI:** 10.1093/jbcr/irad045.236

**Published:** 2023-05-15

**Authors:** Taryn Ketels, Patrick Hosokawa, Arek Wiktor, Elizabeth Kovacs, Juan Idrovo

**Affiliations:** University of Colorado, Denver, Colorado; University Of Colorado, Denver, Colorado; University of Colorado, Aurora, Colorado; University of Colorado, Aurora, Colorado; University of Colorado Denver, Denver, Colorado; University of Colorado, Denver, Colorado; University Of Colorado, Denver, Colorado; University of Colorado, Aurora, Colorado; University of Colorado, Aurora, Colorado; University of Colorado Denver, Denver, Colorado; University of Colorado, Denver, Colorado; University Of Colorado, Denver, Colorado; University of Colorado, Aurora, Colorado; University of Colorado, Aurora, Colorado; University of Colorado Denver, Denver, Colorado; University of Colorado, Denver, Colorado; University Of Colorado, Denver, Colorado; University of Colorado, Aurora, Colorado; University of Colorado, Aurora, Colorado; University of Colorado Denver, Denver, Colorado; University of Colorado, Denver, Colorado; University Of Colorado, Denver, Colorado; University of Colorado, Aurora, Colorado; University of Colorado, Aurora, Colorado; University of Colorado Denver, Denver, Colorado

## Abstract

**Introduction:**

Liver damage following burn injury is associated with a poor prognosis. Thus, prevention, and early identification, of burn-induced liver damage will improve the outcomes of burn patients. Aspartate-aminotransferase (AST) and alanine-aminotransferase (ALT) help detect liver injury in extensive burns >50% of Total body surface area (TBSA). However, their efficacy may be questionable in burns < 50% TBSA. Clinical reports describe albumin to alkaline phosphatase ratio (AAPR) to predict liver injury and dysfunction more accurately than AST and ALT in pathologies like non-alcoholic fatty liver disease and primary liver tumors. However, this ratio has never been tested in burn victims. Herein, we hypothesize that the AAPR may be more useful in detecting burn-induced liver damage. Given that liver biopsies are invasive methods with several complications, we correlated liver histological findings from a burn injury murine model

**Methods:**

Livers and serum were collected from C57BL/6 8-12 weeks female mice, 1, 2, and 4 days after a 15% TBSA scald burn, n=5/group. Histological findings were scored by a pathologist blindly. Serum liver function tests were measured with a colorimetric method. We also conducted a pilot, prospective observational study on 58 burn patients admitted to the Burn Intensive Care Unit (ICU) to follow AST, ALT, and AAPR values. Furthermore, in an initial survival model, we correlated hospital days of stay with the AAPR values

**Results:**

In our murine model of burn injury, liver damage worsens with time. Compared to postburn day 1 the histological damage score significantly increased by 75% at 4 days postburn. AST and ALT were elevated within the first day; however, their values normalized by day 3 postburn, failing to match the progression of liver injury showed by histology. Comparing postburn day 1 the AAPR significantly decreased by 40% on postburn day 4. The enrolled patients were mostly male (74%), with a median age of 50 and an average TBSA of 9.13%. While AST-ALT did not show significant changes in the first 2 weeks after burn, the AAPR on an initial run of a linear model of day predicting ratio decreased over time by -0.08/day (p=0.006). Moreover, our statistical model detected that the likelihood of discharge from ICU increases with the AAPR ratio (Hazard-Ratio 1.18/0.01 increase ratio p =0.003)

**Conclusions:**

AAPR showed a better correlation of burn-induced liver injury in mice than AST and ALT. AAPR decreases in burn patients and may represent postburn liver alterations. AAPR increase raises the likelihood of ICU discharge

**Applicability of Research to Practice:**

Identification and monitoring of liver injury and dysfunction with new modalities may improve the outcome of burn patients

